# Effects of hydration on plasma copeptin, glycemia and gluco-regulatory hormones: a water intervention in humans

**DOI:** 10.1007/s00394-017-1595-8

**Published:** 2017-12-14

**Authors:** Sofia Enhörning, Irina Tasevska, Ronan Roussel, Nadine Bouby, Margaretha Persson, Philippe Burri, Lise Bankir, Olle Melander

**Affiliations:** 1 Department of Endocrinology, Skåne University Hospital, Lund University, Malmö, Sweden; 2 Department of Anesthesiology, Skåne University Hospital, Lund University, Malmö, Sweden; 3 Department of Internal Medicine, Skåne University Hospital, Lund University, Malmö, Sweden; 4 Department of Clinical Sciences, Lund University, Malmö, Sweden; 5 INSERM, Unit 1138, Centre de Recherche des Cordeliers, Paris, France; 6 Université Paris Diderot, Sorbonne Paris Cité, UFR de Médecine, Paris, France; 7 Assistance Publique Hôpitaux de Paris, Hôpital Bichat, DHU FIRE, Paris, France; 8 University Pierre et Marie Curie, Centre de Recherche des Cordeliers, Paris, France; 9 Department of Clinical Sciences, Clinical Research Center, Skåne University Hospital, Jan Waldenströms gata 35, 91:12, 205 02 Malmö, Sweden

**Keywords:** Vasopressin, Glucagon, Insulin, OGTT, Water

## Abstract

**Purpose:**

High plasma copeptin, a marker of vasopressin, predicts diabetes mellitus. We tested if copeptin could be suppressed by increased water intake in healthy individuals, and if a water-induced change in copeptin was accompanied by altered concentrations of glucose, insulin or glucagon.

**Methods:**

Thirty-nine healthy individuals underwent, in random order, 1 week of high water intake (3 L/day on top of habitual intake) and 1 week of normal (habitual) fluid intake (control). Fasting plasma concentrations of copeptin, glucose, insulin and glucagon were compared between the ends of both periods. Furthermore, acute copeptin kinetics were mapped for 4 h after ingestion of 1 L of water.

**Results:**

After acute intake of 1 L water, copeptin was significantly reduced within 30 min, and reached maximum reduction within 90 min with on average 39% reduction (95% confidence interval (95 CI) 34–45) (*p* < 0.001) and remained low the entire test period (4 h). One week of increased water intake led to a 15% reduction (95 CI 5–25) (*p* = 0.003) of copeptin compared to control week. The greatest reduction occurred among subjects with habitually high copeptin and concentrated urine (“water-responders”). Water-responders had significant water-induced reduction of glucagon, but glucose and insulin were unaffected.

**Conclusions:**

Both acute and 1 week extra water intake potently reduced copeptin concentration. In those with the greatest decline (water-responders), who are typically low drinkers with high baseline copeptin, water induced a reduction in fasting glucagon. Long-term trials assessing the effect of water on glucometabolic traits should focus on low-water drinkers with high copeptin concentration.

**Electronic supplementary material:**

The online version of this article (10.1007/s00394-017-1595-8) contains supplementary material, which is available to authorized users.

## Introduction

Vasopressin (VP) is released from the posterior pituitary gland mainly in conditions of increased plasma osmolality or hypovolemia. Apart from maintaining plasma osmolality by mediating water reabsorption in conditions of low water intake, VP has many other physiological functions. VP stimulates hepatic glycogenolysis and gluconeogenesis by acting on VP receptor 1a [1, 2] and release of either insulin or glucagon from the pancreas, depending on the current plasma glucose concentration, through VP receptor 1b (V1bR) [3]. Furthermore, VP plays a role in the hypothalamic–pituitary–adrenal axis by mediating adreno-corticotropic hormone release from the anterior pituitary [4, 5]. Thus, VP may influence glucose homeostasis in many ways [6].

Usual concentrations of VP are very low and the peptide is short lived in plasma. The sensitivity of most VP assays is too low to detect the hormone in the low physiological range [7]. An assay has been developed to indirectly evaluate VP concentration by measurement of copeptin, the C-terminal cleavage product of the VP precursor protein. Copeptin is very stable in vitro and released in a 1:1 ratio with VP [8–10]. We previously showed that fasting plasma concentration of VP, measured as copeptin, strongly predicts new-onset type 2 diabetes [11], a finding later replicated in other large prospective population-based studies [12, 13]. Furthermore, we showed that subjects with high copeptin concentration have an increased risk of all components of the metabolic syndrome [11, 14, 15] as well as cardiovascular disease and premature mortality, both in diabetics and non-diabetics [16–18].

Even though a causal relationship between high VP concentration and risk of diabetes, cardiovascular disease and mortality remains to be proven, there is a growing body of epidemiological and experimental data supporting causality, which in turn has led to increased interest in reducing VP secretion with either pharmacological or non-pharmacological tools. In healthy humans, variation of water intake, even within the normal range, is the most well-established factor controlling release of VP, with low water intake increasing and high water intake decreasing VP secretion, all to keep plasma osmolality constant [19]. Previous experiments have shown that median plasma copeptin values decreased from 3.3 to 2.0 pmol/L within 120 min after an acute water load (20 mL/kg body weight) in young healthy subjects [9]. However, the effect of increased water intake on glucose metabolism and diabetes development has not been studied in humans neither in short-term nor in long-term studies. We recently showed in obese Zucker rats that glucose tolerance deteriorated when they were chronically exposed to high VP. Conversely, when endogenous VP was reduced by an enhanced water intake, their insulin resistance and hepatic fat accumulation were markedly ameliorated [20]. Previous trials and observational studies in humans have demonstrated that high water intake may promote better glucose control, weight loss and decreased cardiovascular risk [21–23]. Furthermore, evidence from humans and animals suggests a protective effect of increased hydration/decreased VP on kidney function [24–26].

The first aim of this study was to test if it is possible to reduce plasma copeptin concentration in healthy individuals, both acutely and within 1 week, by increasing water intake. The second aim was to test if a water-induced reduction in plasma copeptin is accompanied by altered plasma concentrations of glucose, insulin or glucagon, either in the fasted state or during an oral glucose tolerance test (OGTT).

## Subjects and methods

### Study population

Fifty-five healthy subjects aged 20–70 years were recruited via advertisement in local newspaper or through telephone contacts with individuals that have participated in two population-based cohort studies in Malmö [11, 27]. Thirty-nine subjects (71%) completed the study, and 37 subjects had complete data on plasma copeptin concentrations. The participants were exposed to two different intervention procedures in randomized order: water load (acutely and during 1 week), or no change from usual fluid intake (as a time control).

### Study protocol

Each subject underwent two different experimental periods in random order: 1 week with 3 L increased water ingestion per day in addition to each subject’s own food and fluid intake (water week = HWI-Wk), and 1 week on their usual fluid intake (control week = CONT-Wk). Each subject thus served as its own control. During HWI-Wk, the participants were instructed to increase their daily intake of water with 3L and were provided with two bottles (1.5 L each) of still water per day (10 mg/L sodium).

In addition, on day 1 (the first out of seven intervention days), subjects acutely ingested either 1 L of still bottled water (on the HWI-Wk) or only 10 mL of water (on the CONT-Wk) during a maximum time period of 20 min. To map the acute effect of water on copeptin, blood for copeptin measurement was sampled every 30 min for 4 h after the intake of water. For this reason, day 1 of the HWI-Wk then continued with the rest of the daily (3 L) water intake, that is, subjects had to consume 2 additional liters of water on top of their usual food and fluid intakes.

The intervention weeks were separated by 3 weeks of each subject’s usual fluid intake as a wash-out period. The complete study protocol is shown in Fig. 2.

### Laboratory measurements

Copeptin was measured in our lab at baseline in fasting plasma samples stored at − 80 °C using an ultrasensitive assay on KRYPTOR Compact Plus analyzers and a commercial sandwich immunoluminometric assay (ThermoFisher Scientific, B.R.A.H.M.S Biomarkers) as previously described [8, 28]. All other laboratory analyses were performed using certified methods at the University Hospital’s central clinical lab. Procedure for OGTTs: after an overnight fast (no meals or drinks after 10PM the evening before) subjects ingested 75 g of glucose over a maximum period of 3 min, starting sometime between 7:30 and 9:00 AM, followed by blood sampling for glucose measurement at 30, 60 and 120 min. Twenty-four hour urine collections followed procedures developed at the Department of Endocrinology, Skåne University Hospital, and consisted of a comprehensible written instruction aimed at ensuring accurate and complete collection of urine.

### Main outcome measures

On day 1 of the HWI-Wk, plasma copeptin concentrations were measured at 30-min intervals during 4 h after ingestion of 1L water to map copeptin changes after an acute water load.

Absolute differences (“∆values”) between habitual values (mean value of variables measured on days 8 and 9 during CONT-Wk) and post-intervention values (mean value of variables measured on days 8 and 9 during HWI-Wk) were calculated for fasting plasma copeptin, glucose, insulin and glucagon and osmolality. Furthermore, ∆values between habitual (day 9 of CONT-Wk) and post-intervention (day 9 of HWI-Wk) 120-min values during an OGTT were calculated for glucose, insulin and glucagon. Finally, based on the 24 h urine collections returned on day 9, ∆ values between habitual (CONT-Wk) and post-intervention (HWI-Wk) urine osmolality and urine volume were calculated.

### Statistics

Significance of differences between end of HWI-Wk and end of CONT-Wk, as well as differences in copeptin at different times after acute water load compared to time 0 min (pre-water load), was tested using paired *t* test or Wilcoxon signed rank test, depending on normality. Subjects were a posteriori divided into “water-responders” and “non-water-responders” according to the amplitude of the copeptin decline (∆copeptin) between the CONT-Wk and the HWI-Wk. Water-responders were defined as subjects in the top tertile of the copeptin decline. Significance of differences between these two subgroups was tested using independent sample *t* test or Mann–Whitney *U* test depending on normality. We used linear regression analysis of crude and serum albumin-corrected residuals between water-induced changes (end of HWI-Wk vs end of CONT-Wk) of copeptin vs changes of glucose, insulin and glucagon during the same time period.

SPSS statistical software version 23 (SPSS Inc., Chicago, Ill., USA) was used for all analyses. A two-sided *p* value of < 0.05 was considered statistically significant.

## Results

The 37 participants had a median age of 53 year (interquartile range 37–68). Nine were men and mean body mass index was 25.2 kg/m^2^ (SD 4.4).

### Acute and 1-week effect of increased water intake on copeptin

**Fig. 1:**
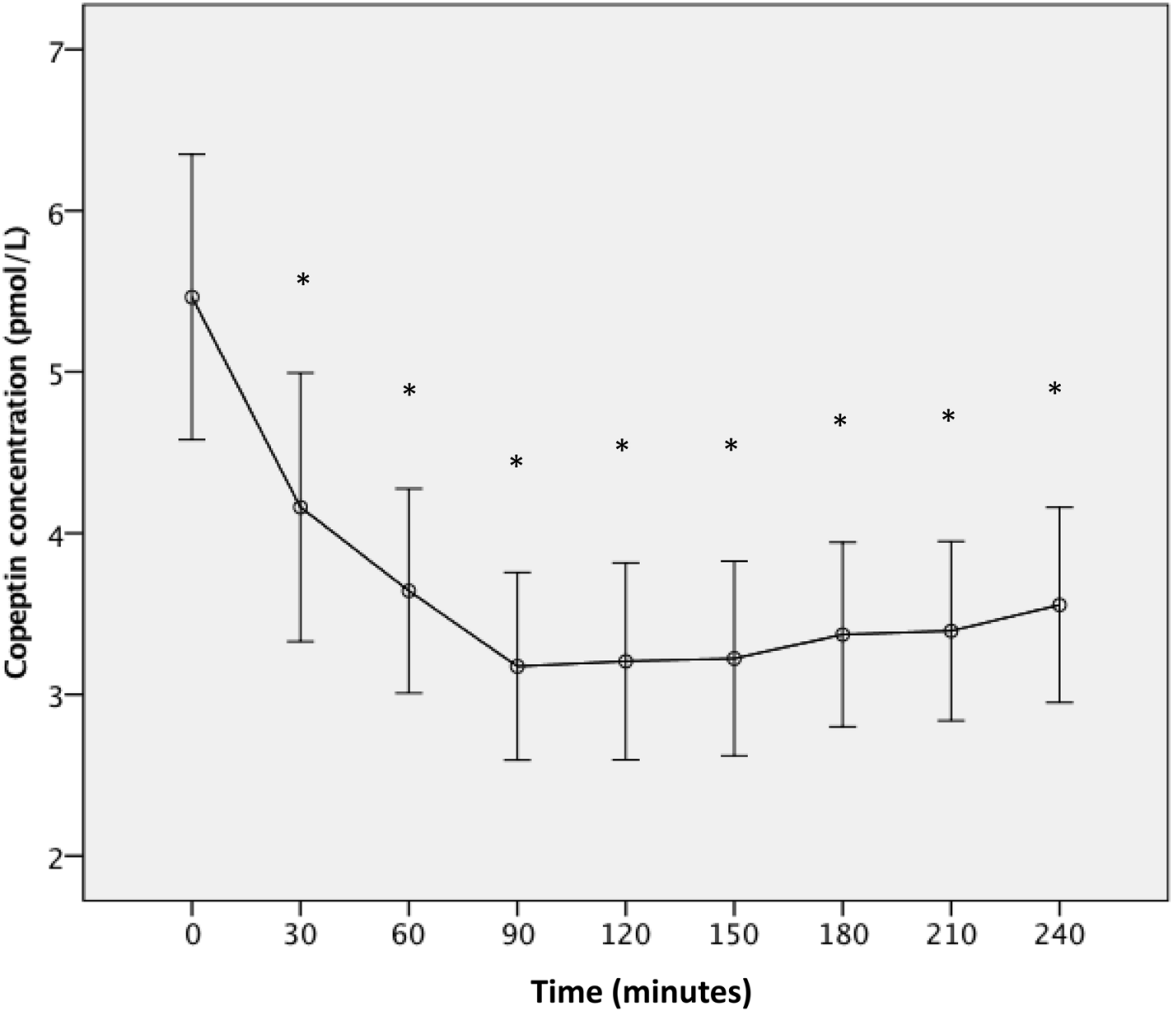
Effect of acute water load on plasma copeptin. Plasma copeptin concentration [expressed as mean (95 CI)] measured minutes after 1 L water intake (*n* = 39). At 0 min, median (IQ) copeptin value is 5.05 (3.53–6.44) pmol/L, whereas it decreases to 2.77 (2.28–3.57) pmol/L at 90 min. *p < 0.001

After a rapid oral water load of 1 L, plasma copeptin was significantly reduced within 30 min, and reached maximum reduction within 90 min with on the average 39% reduction (95 CI 34–45; *p* < 0.001). This significant reduction of copeptin was sustained over the entire duration of the test (4 h) (Fig. 1).

One week of increased water intake was accompanied by a 15% reduction (95 CI 5–25; *p* = 0.003) of copeptin at the end of the HWI-Wk compared to that at the end of the CONT-Wk (Table 1).

**Table 1:** Blood and urine parameters at the end of control week and the end of water week (*n* = 37)

	End of control week	End of water week	Δ change^a,b^	*p* value
P-copeptin (pmol/L)	5.33 (3.45–7.14)	3.95 (3.14–5.32)	1.22 (1.75)	< 0.001
U-osmolality (mosm/kg H_2_O)	493 (372–680)	232 (196–297)	290 (218)	< 0.001
U-volume (mL/24 h)	1361 (1050–1821)	3531 (3031–3935)	− 1947 (962)	< 0.001
Osmolar excretion rate (mosm/24 h)	690 (510–849)	820 (676–967)	− 91 (172)	0.003
P-osmolality (mosm/kg H_2_O)	294.0 (291.3–297.0)	293.5 (289.3–295.5)	2.01 (5.25)	0.03
P-urea (mmol/L)^b^	4.74 (1.12)	4.22 (0.81)	0.52 (0.86)	0.001
P-sodium (mmol/L)^b^	140.4 (1.61)	140.3 (1.60)	0.19 (1.67)	0.50
P-potassium (mmol/L)^b^	3.83 (0.18)	3.86 (0.18)	− 0.03 (0.18)	0.26
P-creatinine (µmol/L)^b^	73.9 (11.3)	74.0 (12.6)	− 0.43 (5.13)	0.61

Data are expressed as median (interquartile range) if nothing else is specified

Fasting values if nothing else is specified

^a^Δchange = end of control week − end of water week

^b^Data are expressed as mean (SD)

The amplitude of copeptin reduction at the end of HWI-Wk vs after CONT-Wk was strongly and positively correlated with higher habitual copeptin (*r* = 0.63, *p* < 0.001), higher habitual urine osmolality (*r* = 0.62, *p* < 0.001) and lower habitual urine volume (*r* = − 0.52, *p* = 0.001), i.e., indices of lower water intake during the habitual (CONT-Wk) state. Water-responders, i.e., subjects belonging to the top tertile of water-induced copeptin reduction (*n* = 12), had an average copeptin reduction of 41% (95 CI 34–49; *p* < 0.001), whereas the remaining subjects (non-water-responders, *n* = 25) showed a non-significant reduction of copeptin of 2.7% (95 CI − 8.3 to 14; *p* = 0.61) (Table 2). In line with the continuous correlation analyses, the main characteristics separating water-responders from non-water-responders were that water-responders had habitually higher copeptin, higher urine osmolality and lower urine volume, i.e., indices of being less hydrated (Table 2; Fig. 3a, b).

**Table 2:** Water-induced reduction of copeptin and habitual blood and urine parameters (at the end of control week) in water-responders and non-water-responders

	Non-water-responders^a^ (*n* = 25)	Water-responders^b^ (*n* = 12)		*p* value
Reduction in plasma copeptin concentration (after 1 week of extra water as compared to after control week)				
P-copeptin reduction (pmol/L)	0.24 (− 0.21–0.69)^c^	3.26 (2.71–3.80)^c^		< 0.001
P-copeptin reduction (% of habitual copeptin concentration)	2.7 (− 8.3–13.7)^c^	41.2 (33.8–48.6)^c^		< 0.001
Habitual urine and plasma concentrations (at the end of control week)				
P-copeptin (pmol/L)	3.64 (3.22–5.52)	7.46 (6.02–9.53)		< 0.001
U-osmolality (mosm/kg H_2_O)	455.0 (316.5–562.5)	664.0 (473.5–962.8)		0.008
U-volume (mL/24 h)	1411 (1280–2194)	1036 (768–1357)		0.005
Osmolar excretion rate (mosm/24 h)	714 (544–789)	635 (482–1001)		0.44
P-osmolality (mosm/kg H_2_O)	293.0 (290.0–296.5)	295.0 (293.5–301.5)		0.04
P-urea (mmol/L)^d^	4.53 (0.94)	5.19 (1.44)		0.18^e^
P-sodium (mmol/L)^d^	140.1 (1.52)	141.4 (1.58)		0.03^e^
P-potassium (mmol/L)^d^	3.81 (0.18)	3.85 (0.18)		0.52^e^
P-creatinine (µmol/L)	71.0 (65.0–83.0)	79.5 (66.0–87.0)		0.79

Data are expressed as median (interquartile range) if nothing else is specified

Fasting values if nothing else is specified

^a^Non-water responder refers to subjects with the lowest water-induced copeptin reduction, i.e., first and second tertile of Δ-copeptin (corresponding to a copeptin reduction of ≤ 2 pmol/L)

^b^Water responder refers to subjects with the highest water-induced copeptin reduction, i.e., third tertile of Δ-copeptin (corresponding to a copeptin reduction of > 2 pmol/L)

^c^Data are expressed as mean (95 CI)

^d^Data are expressed as mean (SD)

^e^Independent sample *T* test

### Effects of 1 week of increased water intake on plasma glucose, insulin and glucagon concentrations

**Fig. 2:**
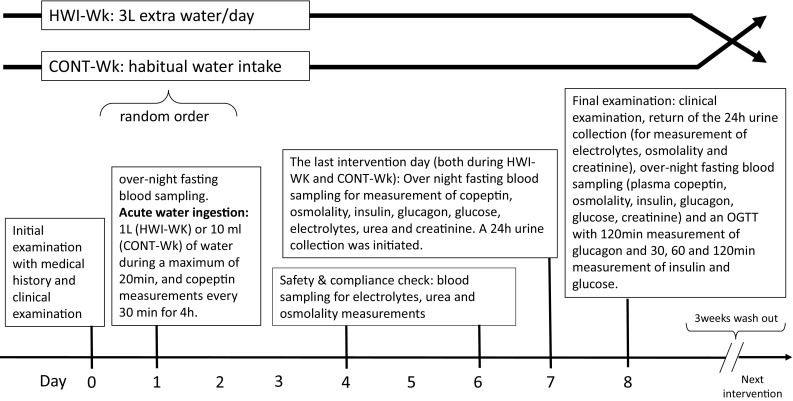
Study protocol. All blood and urine samplings were carried out both during HWI-Wk and CONT-Wk

**Fig. 3:**
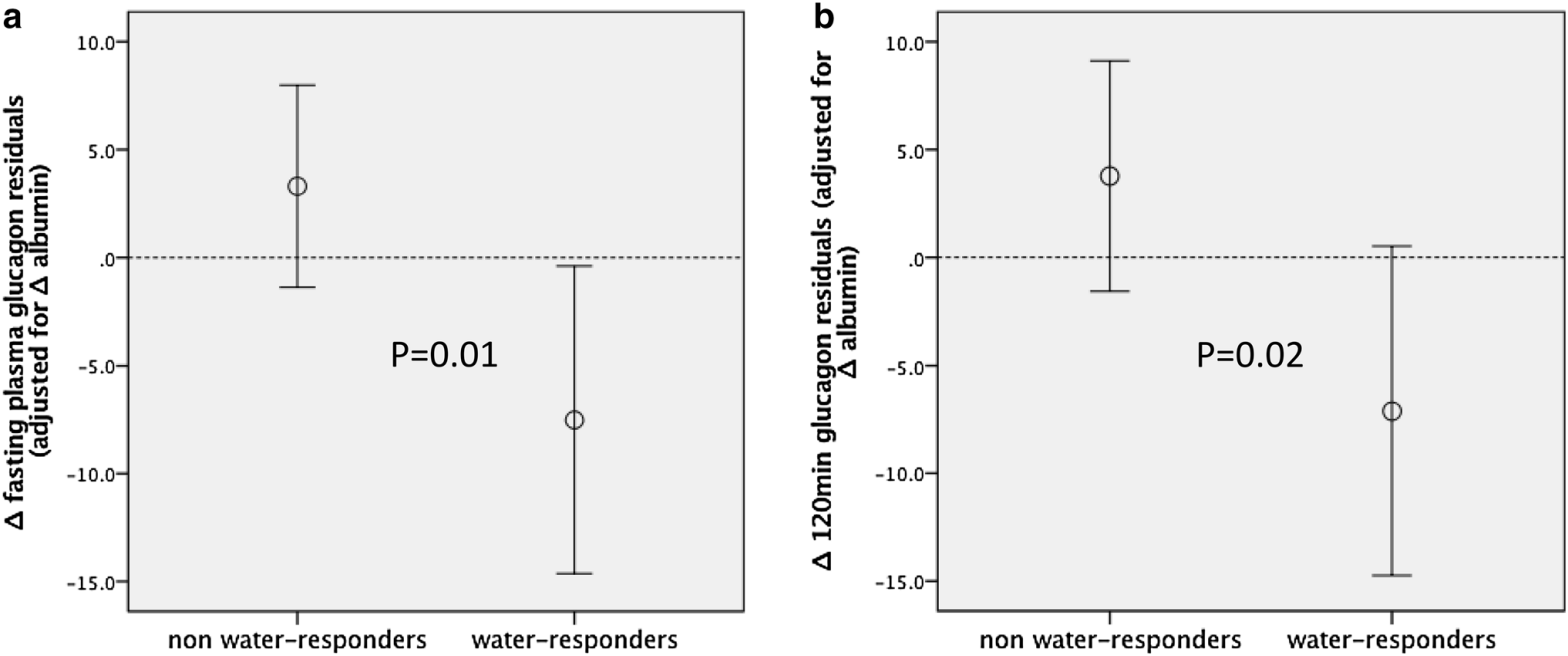
**a, b** Fasting and 120 min post-OGTT glucagon reduction in non-water-responders and water-responders, respectively. Glucagon reduction is expressed as mean (95 CI) of Δfp-albumin adjusted Δglucagon residuals. Δ = difference between concentration at the end of HWI-Wk and end of CONT-Wk. Water-responders refer to subjects with the highest water-induced copeptin reduction, i.e., third tertile of Δcopeptin (corresponding to a copeptin reduction of > 2 pmol/L)

Overall, there were no significant differences in glucose, insulin or glucagon concentrations at the end of HWI-Wk as compared to end of CONT-Wk (Table 3), nor was there any significant correlation between ∆copeptin and 0 min ∆glucose (*r* = − 0.08, *p* = 0.6), 120 min∆ glucose (*r* = 0.02, *p* = 0.9), 0 min ∆insulin (*r* = − 0.15, *p* = 0.39) or 120 min ∆ insulin (*r* = − 0.16, *p* = 0.36). Results were similar after correction for change of water-induced plasma albumin as a proxy for water-induced plasma dilution. However, greater water-induced reduction of copeptin significantly associated with reduction of glucagon (∆glucagon) both at 0 and 120 min of an OGTT when going from habitual water intake to high water intake [crude correlations between ∆copeptin and 0 min ∆glucagon (*r* = 0.37, *p* = 0.03), and 120 min post-OGTT ∆glucagon (*r* = 0.39, *p* = 0.02), respectively]. To make sure that water-induced reductions of glucagon were not simply a result of volume expansion, we corrected these correlations for water-induced change of plasma albumin concentration. The correlations remained significant (*p* = 0.01 for water-induced glucagon reduction at 0 min and *p* = 0.02 at 120 min of the OGTT). In concert with this, the water-induced change of both fasting and 120 min glucagon differed significantly between water-responders and non-water-responders (Fig. 3a, b). Moreover, in water-responders, high water intake was accompanied by a significant decrease of glucagon under fasting conditions (*p* = 0.04) and a borderline significant glucagon decrease at 120 min of an OGTT (*p* = 0.07), whereas this was not the case among non-water-responders (Fig. 2a, b).

**Table 3:** Glucometabolic parameters at the end of control week and the end of water week (*n* = 37)

	End of control week	End of water week	Δchange^a, b^	*p* value
P-glucose (mmol/L)^b^	5.44 (0.45)	5.54 (0.55)	− 0.08 (0.41)	0.23
P-glucose 120 min (mmol/L)^b,c^	5.87 (1.80)	5.62 (1.88)	0.24 (1.58)	0.35
Glucagon (pmol/L)	35.3 (29.1–45.1)	35.8 (29.3–49.5)	− 0.42 (11.9)	0.84
Glucagon 120 min (pmol/L)^c^	30.5 (26.3–38.5)	30.5 (25.0–44.3)	− 0.42 (12.7)	0.85
Insulin (mIE/L)	7.75 (4.50–11.00)	8.00 (5.00–11.13)	0.40 (2.2)	0.29
Insulin 120 min (mIE/L)^c^	32.0 (22.0–60.5)	28.0 (15.0–52.0)	4.39 (19.4)	0.18

Data are expressed as median (interquartile range) if nothing else is specified

Fasting values if nothing else is specified

^a^Δchange = end of control week − end of water week

^b^Data are expressed as mean (SD)

^c^During an oral glucose tolerance test (OGTT)

Habitual glucometabolic parameters did not differ significantly between water-responders and non-water-responders (Supplemental table 1).

## Discussion

The key finding of the present study is that in 37 healthy volunteers there is a significant reduction of copeptin after an increased water intake for 1 week, as compared with habitual water intake. However, the effect varies substantially between individuals. The one-third showing the largest copeptin reduction (water-responders) were characterized by indices of relatively low habitual water intake, as compared to non-water-responders. One week of increased hydration does not alter glycemia, insulin or glucagon concentrations in the whole group. However, in water-responders it leads to a significant reduction of fasting glucagon concentration.

An acute water load results in a potent approximately 40% reduction of copeptin, which is sustained at least over 4 h.

We and others previously established that VP, as measured by copeptin, is an independent risk factor for diabetes, the metabolic syndrome, chronic kidney disease, cardiovascular disease and premature death in the population [11, 15–17, 29, 30]. It is well known that certain diseases such as heart failure, acute myocardial infarction, hemorrhage and sepsis result in marked elevation of copeptin [31–34], but, in the general population, the most likely cause of having elevated copeptin is a low water intake. Given the multiple reports on an independent relationship between high copeptin and risk of cardiometabolic diseases, the potential of using increased hydration as a preventive tool for these diseases has acquired increasing interest. Importantly, in animal models, beneficial effects on metabolism have been demonstrated through VP reduction achieved by increased hydration, whereas elevation of VP deteriorated glucose tolerance, pointing at a likely causal relationship [20]. Furthermore, genetic variation in the human VP gene was recently associated with both elevated copeptin and increased risk of hyperglycemia, providing additional support of causality between elevated copeptin and metabolic disease [35]. If causal, this relationship would open the possibility of a metabolic intervention based on water intake. However, it is not known to what extent an increased water intake is instrumental in decreasing VP secretion. The first aim of this study was, therefore, to test if, and to what extent, copeptin can be reduced in healthy humans by increased water intake acutely and over 1 week.

The acute effect of a large oral water load on copeptin has been previously demonstrated once in younger subjects [9]. What is new and important from a therapeutic point of view is that the copeptin reduction was sustained throughout the 4 h of the test and, judging from the curve shape, probably lasted even longer (Fig. 1). This suggests that water does not have to be continuously ingested to achieve sustained reduction of copeptin, but that the same amount can be drunk during a short period of time (> 20 min) with a sustained effect over at least 4 h. When investigating the effect of increased water intake on copeptin over 1 week, we compared copeptin after HWI-Wk to that after CONT-Wk. Although the subjects were instructed to ingest 3 L of water on top of habitual intake, the difference in urine volume after the two respective weeks suggests that the achieved difference in water intake was close to 2 L per day (Table 1). Thus, it seems likely that when adding 3 L of water, the habitual intake decreases.

The 15% average reduction of copeptin was largely driven by the water-responders, who had an average copeptin reduction of 41%, compared to virtually no reduction at all in non-water-responders (Table 2). To characterize the water-responders, i.e., individuals who would benefit the most from increased water used therapeutically to decrease VP, we compared measures of hydration during habitual water intake (end of CONT-Wk) between water-responders and non-water-responders (Table 2). This comparison showed that water-responders are characterized by higher copeptin, higher urine osmolality and lower urine volume, i.e., indices of relatively lower water intake. This suggests that any intervention study aiming at lowering VP by increasing water intake should focus on subjects with high copeptin and low water intake.

As high copeptin has been repeatedly shown to be a strong independent risk factor for diabetes, the second aim of our study was to investigate if a reduction of copeptin by increased water intake for 1 week may influence glycemia, insulin or glucagon concentrations. We did not find any difference in these metabolic indices at the end of the HWI-Wk compared to the end of the CONT-Wk. It is possible that 1 week is too short to lead to metabolic alterations reflected by these measures, or that the study was underpowered to detect an existing effect. One additional explanation for the overall neutral effects on these metabolic factors could be that metabolic alterations are only seen in subjects whose copeptin is in fact reduced by increased water intake, i.e., in water-responders. Interestingly, whereas glycemia and insulin were not altered by hydration in water-responders, fasting glucagon was significantly reduced, and glucagon post-oral glucose challenge was borderline significantly reduced (Fig. 3a, b). Although this finding needs replication, it suggests that just 1 week of increased hydration in subjects with habitual low water intake leads to a marked reduction of VP which is paralleled by a reduction of glucagon. High glucagon secretion is an important risk factor for impaired glucose tolerance and type 2 diabetes [36]. Type 2 diabetes is associated with elevated glucagon concentration throughout the day [37], and both type 2 diabetes and impaired glucose tolerance are associated with impaired suppression of glucagon secretion [38, 39]. Furthermore, elevated glucagon secretion is manifest long before the onset of impaired glucose tolerance [39]. VP stimulates glucagon secretion by activation of V1bR in α-cells of pancreatic islets [3] which is concordant with our finding that fasting glucagon concentration was reduced upon water-induced suppression of VP (copeptin). In addition, we recently showed in rodents that during conditions of high VP, selective pharmacological blockade of V1bR with SR149415 resulted in reduction of plasma glucagon [40]. Taken together, our finding that water-induced decrease of copeptin in water-responders is associated with fasting glucagon reduction encourages long-term studies of anti-diabetic effects of water supplementation in subjects with low water intake.

We previously showed that the risk of future diabetes development among normoglycemic subjects was 3.5-fold higher in the top quartile compared to the bottom quartile of fasting plasma copeptin concentration after adjustment for known diabetes risk factors. The top quartile corresponded to copeptin of > 6.1 pmol/L in females and > 10.7 pmol/L in males [11]. In the current study population, a similar proportion of subjects (23%) had habitual fasting plasma copeptin concentration above these thresholds, denoting high diabetes risk, and 89% of these high-diabetes-risk subjects were water-responders. We, therefore, suggest that approximately 25% of the population would represent an ideal target group for studying the effects of water supplementation on diabetes risk, as these subjects, apart from having a high diabetes risk, are, to the great majority, water-responders with low habitual water intake.

### Limitations

We did not control the participants’ food intake in the current study. Osmolar excretion was significantly higher at the end of HWI-Wk than at the end of CONT-Wk (Table 1). This difference obviously results from a greater food intake during increased hydration. We previously observed that rats tend to eat more when hydration is increased [24, 41]. It is likely that in the present study, copeptin concentration could have been reduced even more during HWI-Wk if food intake had not increased, because increased protein intake is known to increase VP secretion. However, the habitual osmolar excretion rate was similar in water-responders and non-water-responders (Table 2) and there was no significant difference in ∆osmolar excretion (CONT-Wk − HWI-Wk) between water-responders and non-water-responders (*p* = 1.0). Thus, the better glucagon-lowering effect observed in water-responders (Fig. 3a, b) cannot result from differences in the amount of ingested food. Furthermore, we did not monitor the participants’ actual water intake with questionnaires or diaries in the current study. Instead, we used measures of urinary volume as a proxy for water intake. Finally, the choice to set the increased water ingestion to 3 L per day was arbitrary.

## Conclusion

High water intake acutely leads to a potent and sustained reduction of plasma copeptin. Over 1 week, the copeptin lowering effect of increased water intake is on the average more modest. However, in subjects with habitually high copeptin and signs of low water intake (i.e., water-responders), the reduction in copeptin is about 40%. Furthermore, water-responders exhibit reduced concentrations of glucagon. Our results indicate that water-responders, who have both greater diabetes risk and markedly reduced copeptin after high water intake, represent an ideal target segment of the healthy population for a long duration randomized controlled trial testing the effects of hydration on cardiometabolic outcomes.

## Electronic supplementary material

Below is the link to the electronic supplementary material.

Supplementary material 1 (DOCX 11 KB)
