# Supplementary material for: Effects of hydration on plasma copeptin, glycemia and gluco-regulatory hormones: a water intervention in humans
Source: Eur J Nutr. 2017 Dec 14;58(1):315–24. doi: 10.1007/s00394-017-1595-8 (PMC6424930; doi:10.1007/s00394-017-1595-8)
Supplement: Supplementary file 1 — Supplementary material 1 (DOCX 11 KB) [file 394_2017_1595_MOESM1_ESM.docx]

|  | Non water-responders ^a^  (n=25) | Water-responders ^b^  (n=12) | P-value |
| --- | --- | --- | --- |
| P-glucose (mmol/L) | 5.35 (5.10-5.73) | 5.40 (5.20-5.65) | 0.71 |
| P-glucose 120 min (mmol/L) ^c^ | 5.70 (4.20-6.75) | 5.75 (4.90-6.50) | 0.55 |
| Glucagon (pmol/L) | 32.0 (29.1-41.3) | 39.5 (29.0-50.5) | 0.20 |
| Glucagon 120 min (pmol/L) ^c^ | 30.0 (25.0-32.0) | 36.0 (27.8-41.8) | 0.10 |
| Insulin (mIE/L) | 7.00 (4.25-10.50) | 10.00 (7.13-13.00) | 0.13 |
| Insulin 120 min (mIE/L) ^c^ | 27.0 (22.0-49.5) | 44.0 (17.0-62.0) | 0.49 |
| Data are expressed as median (interquartile range).  Fasting values if nothing else specified.  ^a^ Non water responder refers to subjects with the lowest water-induced copeptin reduction, i.e. first and second tertile of Δ-copeptin (corresponding to a copeptin reduction of ≤ 2pmol/L).  ^b^ Water responder refers to subjects with the highest water-induced copeptin reduction, i.e. third tertile of Δ-copeptin (corresponding to a copeptin reduction of > 2pmol/L).  ^c^ During an oral glucose tolerance test (OGTT). | | | |

Supplemental table 1. Habitual glucometabolic parameters (at the end of control week) in water-responders and non water-responders
